# The first description of a hormone‐sensitive lipase from a basidiomycete: Structural insights and biochemical characterization revealed *Bjerkandera adusta Ba*EstB as a novel esterase

**DOI:** 10.1002/mbo3.463

**Published:** 2017-03-01

**Authors:** María del Rayo Sánchez‐Carbente, Ramón Alberto Batista‐García, Ayixón Sánchez‐Reyes, Angela Escudero‐Garcia, Catalina Morales‐Herrera, Laura I. Cuervo‐Soto, Leidys French‐Pacheco, Arline Fernández‐Silva, Carlos Amero, Edmundo Castillo, Jorge Luis Folch‐Mallol

**Affiliations:** ^1^ Centro de Investigación en Biotecnología Universidad Autónoma del Estado de Morelos Cuernavaca Morelos Mexico; ^2^ Centro de Investigación en Dinámica Celular Instituto de Investigación en Ciencias Básicas y Aplicadas Universidad Autónoma del Estado de Morelos Cuernavaca Morelos Mexico; ^3^ Departamento de Biología Facultad de Ciencias Universidad Antonio Nariño Bogota Colombia; ^4^ Centro de Investigaciones Químicas Instituto de Ciencias Básicas y Aplicadas Universidad Autónoma del Estado de Morelos Cuernavaca Morelos Mexico; ^5^ Instituto de Biotecnología Universidad Nacional Autónoma de México Cuernavaca Morelos Mexico

**Keywords:** *Bjerkandera adusta*, ergosterol esters, esterase, Hormone‐Sensitive Lipase

## Abstract

The heterologous expression and characterization of a Hormone‐Sensitive Lipases (HSL) esterase (*Ba*EstB) from the Basidiomycete fungus *Bjerkandera adusta* is reported for the first time. According to structural analysis, amino acid similarities and conservation of particular motifs, it was established that this enzyme belongs to the (HSL) family. The cDNA sequence consisted of 969 nucleotides, while the gene comprised 1133, including three introns of 57, 50, and 57 nucleotides. Through three‐dimensional modeling and phylogenetic analysis, we conclude that *Ba*EstB is an ortholog of the previously described *Rm*EstB‐HSL from the phylogenetically distant fungus *Rhizomucor miehei*. The purified *Ba*EstB was characterized in terms of its specificity for the hydrolysis of different acyl substrates confirming its low lipolytic activity and a noticeable esterase activity. The biochemical characterization of *Ba*EstB, the DLS analysis and the kinetic parameters determination revealed this enzyme as a true esterase, preferentially found in a dimeric state, displaying activity under alkaline conditions and relative low temperature (pH = 10, 20°C). Our data suggest that *Ba*EstB is more active on substrates with short acyl chains and bulky aromatic moieties. Phylogenetic data allow us to suggest that a number of fungal hypothetical proteins could belong to the HSL family.

## 1 Introduction

Esterases (carboxylic ester hydrolases, EC 3.1.1) including lipases (triacylglycerol hydrolases, EC 3.1.1.3.) are important biocatalysts that mediate stereospecific hydrolysis, transesterification and a variety formation of primary and secondary alcohols (Akoh, Lee, Liaw, Huang, & Shaw, 2004; Lopes, Fraga, Fleuri, & Macedo, 2011). Due to their particular properties such as broad substrate specificity, high chemo‐, regio‐ and stereoselectivity and generally with no requirement for cofactors, these enzymes have a wide variety of industrial uses such as the production of detergents, pharmaceuticals, cosmetics, and paper as well as in biodiesel industry among others (Andualema & Gessesse, 2012; Bornscheuer, 2002; Carrière et al., 1998; Chahinian & Sarda, 2009). While lipases act on water‐insoluble substrates, such as long‐chain triglycerides, esterases hydrolyze preferentially ‘simple’ esters and usually only triglycerides bearing short‐chain fatty acids (<C_10_) (Jaeger, Dijkstra, & Reetz, 1999). Most of lipases can also be distinguished from esterases by the phenomenon of interfacial activation, where the enzymes are active at the interface between their hydrophobic lipid substrate and the hydrophilic medium (oil–water interface) (Fojan, Jonson, Petersen, & Petersen, 2000).

Esterases and lipases have been isolated from microorganisms, plants, animals, and metagenomes (Fojan et al., 2000; Gopinath, Anbu, Lakshmipriya, & Hilda, 2013). They have also been produced in heterologous hosts upon bioinformatics screening of public databases of genomes (Barriuso, Prieto, & Martínez, 2013). There is still interest in the characterization of microbial lipolytic enzymes and esterases because of their high production yields and the easy genetic manipulation of the microorganisms compared with other organisms. Both enzymes share the α/β fold in their structure and most of them present the consensus sequence GXSXG as a signature motif. According to amino acid similarities and conservation of particular motifs, lipolytic enzymes (including esterases) are classified into C, H, X, and L blocks (Lenfant et al., 2013).

Block H includes Hormone‐Sensitive Lipases (HSL), where the majority of the enzymes resulted to be esterases (Ali, Verger, & Abousalham, 2012; Huang et al., 2016; Li et al., 2015). In mammals, HSL have been proposed as esterases that hydrolyze diacylglycerol for the generation of ATP and cholesteryl esters precursors to deliver cholesterol for the synthesis of steroid hormones (Kraemer, 2007). In fungi, ergosterol is used instead of cholesterol to maintain fluidity, permeability and integrity of the plasma membrane and adequate function of membrane‐bound proteins (Holick, 2003; Sun, Gao, Ling, & Lou, 2005). Ergosteryl esters are found in lipid particles in the cytoplasm and when unesterified may be used as a source for ergosterol in membrane synthesis (Shobayashi et al., 2005; Zweytick, Athenstaedt, & Daum, 2000).

Bacterial lipolytic enzymes are classified in eight families, where proteins with high similarity to mammalian HSL belong to family IV (Arpigny & Jaeger, 1999). Based on massive sequence alignment and different conserved motifs, two subfamilies of bacterial HSL have been proposed, the G**T**S**A**G motif subfamily and the G**D**S**A**G motif subfamily, where the labeled amino acids are defined as arbitrary residues in the pentapeptide conserved motif G**X**S**X**G (Jeon et al., 2012; Li et al., 2014).

Lipolytic enzymes from fungi have been extensively characterized from *Candida antarctica, C. rugosa,* and from filamentous fungi as *Aspergillus niger*,* Rhizopus orizae*,* Penicillium camembertii* among others, that have been commercialized and used in dairy, oil, and fat industries (Anobom et al., 2014; Borrelli & Trono, 2015; Houde, Kademi, & Leblanc, 2004). However, only two fungal lipolytic enzymes from the HSL family (*Rm*EstA and *Rm*EstB) have been studied (*Rm*:* Rhizomucor miehei*, Est: Esterase). Both of them were identified from the mucoral thermophilic fungus *R. miehei*. Interestingly, *Rm*EstA and *Rm*EstB exhibit distinct substrate specificities: *Rm*EstA shows high activity toward long‐chain esters, whereas *Rm*EstB favors hydrolysis of short‐chain esters (Liu et al., 2013; Yan et al., 2014; Yang, Qin, Duan, Yan, & Jiang, 2015). In spite of these reports, there is still a lack of information of lipolytic enzymes from the HSL family in fungi and their role is uncertain.

*Bjerkandera adusta* is a “white‐rot” Basidiomycete well known for its high ligninolytic activities (Romero, Speranza, García‐Guinea, Martínez, & Martínez, 2007; Wang, Vazquez‐Duhalt, & Pickard, 2003), which are attractive to biorefinery and bioremediation fields. Few reports have described its extracellular enzymatic activities from crude extracts (Bancerz & Ginalska, 2007; Quiroz‐Castañeda, Pérez‐Mejía, Martínez‐Anaya, Acosta‐Urdapilleta, & Folch‐Mallol, 2011; Quiroz‐Castañeda et al., 2009). Only two *B. adusta*′s genes from strain UAMH‐8258 have been cloned and overexpressed in heterologous systems and their proteins characterized: a loosenin (an expansin‐like protein) (Quiroz‐Castañeda, Martínez‐Anaya, Cuervo‐Soto, Segovia, & Folch‐Mallol, 2011) and a carbohydrate esterase (Cuervo‐Soto et al., 2015). Interestingly, a report in 2005 showed that *B. adusta* R59 degrades highly recalcitrant xenobiotics such as the anthraquinonic antibiotic daunomycin and other humic acids. Among the enzymatic activities secreted by the fungus to degrade the xenobiotics were laccases, peroxidases, cellulases, hemicellulases, and lipolytic enzymes. Moreover, lipolytic activity was stimulated in the presence of humic acids (Belcarz, Ginalska, & Kornillowicz‐Kowalska, 2005). However, the study did not show data of purified enzymes.

In this work, we report the characterization of the first HSL esterase reported from a Basidiomycete (*Ba*EstB). The cDNA was obtained from a *B. adusta* library grown on crude oil. Blastp results showed high similarity with esterase family IV that includes HSL members. Classification within the HSL family was confirmed through phylogenetic analysis and homology modeling of *Ba*EstB. The esterase activity was confirmed by the preference of the enzyme for shorter chain substrates and the lack of activity on a rhodamine triglyceride assay.

## 2 Materials and Methods

### 2.1 *Ba*EstB modeling, structural alignment, three‐dimensional superposition and structure‐based and sequence‐based phylogenies

Upon PSI‐BLAST (Position‐Specific Iterated) analysis of 768 sequenced clones from a cDNA library form *B. adusta*, we identified a sequence with homology to the α/β hydrolase Esterase/Lipase superfamily. The Open Reading Frame (ORF) in this sequence was named as *Ba*EstB (GenBank accession number KX580963). The complete amino acid sequence of the *Ba*EstB was submitted to the I‐TASSER server (Roy, Kucukural, & Zhang, 2010; Yang, Qin, et al., 2015; Yang, Yan, et al., 2015; Zhang, 2008) without constraints in order to get a three‐dimensional model. A second modeling round was performed using PDB (Protein Data Bank) 4WY8 and 4ZRS as templates, since these PDBs were the best templates found during the first modeling round. Structural alignments and three‐dimensional superpositions were obtained considering the PDBs identified by I‐TASSER as close structural neighbors in order to identify the cap and catalytic domains, the catalytic triad, the conserved motifs, and other residues involved in the catalysis of *Ba*EstB. The model visualization and structural alignment, including the Root Mean Square Deviation (RMSD) values, were obtained in Visual Molecular Dynamic program (VMD) (Humphrey, Dalke, & Schulten, 1996).

A structure‐based phylogenetic tree according with the RMSD derived from *Ba*EstB's structural comparison with its closest structural analogs was prepared in VMD and visualized in Phylogeny.fr server (http://www.phylogeny.fr/). A second phylogenetic tree (amino acid sequence‐based reconstruction) was obtained on line in the same server in order to describe the relationships of *Ba*EstB with related sequences obtained from the Blast results. Phylogeny.fr considers various bioinformatics algorithms to construct a robust phylogenetic tree from a set of sequences (Dereeper, Audic, Claverie, & Blanc, 2010; Dereeper et al., 2008); for the generation of phylogenetic trees, MUSCLE was used for the multiple alignments, Gblocks for the automatic alignment curation (in order to eliminate poorly aligned positions, not allowing smaller final blocks and less strict flanking positions), *PhyML,* for tree building and TreeDyn for tree drawing (Dereeper et al., 2008). The Maximum Likelihood method was used to estimate the phylogenetic tree; the branch support was assessed using the *ALTr* algorithm and the Jones‐Thornton‐Taylor (JTT) model was used to estimate distances for amino acids (Dereeper et al., 2008). The parameters used during the MUSCLE alignment were those recommended by the Phylogeny.fr platform (custom mode with 16 as the maximum number of iterations). A lipase from *Candida cylindracea* (gi: 1325988) was used as an outgroup.

### 2.2 Cloning of *Ba*EstB

The coding sequence of the *Ba*EstB was amplified using primers Forw*Ba*EstB (5′gaattcatggaatctatccgtctgtc3′) and Rev*Ba*EstB (5′tctaga **cc**ctattccgtcgctggta3′) with cutting sites underlined for *Eco*RI and *Xba*I, respectively, which allowed the subcloning into the expression vector (pPICZαA); the two cytosines in bold were added to put the sequence in frame with the myc‐ and poly‐His tags. The PCR conditions were as follows: 95°C 5 min (one cycle); 95°C 45 s, 55°C 60 s and 72°C 2 min (30 cycles) and a final extension step of 72°C 7 min (one cycle), on a thermocycler (Axigen‐Maxigene) and the amplification was carried out with *Pfu* DNA polymerase (Jena Biosciences). The amplified product was purified with the Column DNA Gel Extraction Spin Kit (Thermo Scientific), ligated into vector pJET (pJET‐*Ba*EstB) and transformed into *E. coli* DH5α electrocompetent cells for subsequent sequencing.

The genomic sequence of *Ba*EstB was obtained using the Genome Walker Kit (Clontech) following the manufacturer's instructions using oligonucleotides pDNRlib Fwd: 5′ ACCATGGAATCTATCCGTCT 3′ and pDNRlib Rev: 5′ AGAAAATGTCATTACGGTGG 3′. The PCR conditions were as follows: 95°C‐5 min (one cycle), 95°C 1 min; 57°C 30 s; 73°C 2 min (30 cycles); and a final extension step of 73°C for 5 min, using the same apparatus and enzyme as described above. The amplified fragments were purified as described above for sequencing.

### 2.3 Expression of *Ba*EstB in *Pichia pastoris* X‐33 and enzyme purification

pJET‐*Ba*EstB was digested with *Eco*RI and *Xba*I, and the released *Ba*EstB cDNA fragment was inserted into pPICZαA (Invitrogen, USA) digested with the same enzymes. This construct was designated as pPICZαA/*Ba*EstB and was transformed into *E. coli* DH5α. Restriction digestion and DNA sequencing verified the identity of the insert. This construction was linearized with *Sac*I, and *P. pastoris* X‐33 cells were transformed by electroporation for 5 ms at 2,000 volts employing an Eporator apparatus (Eppendorf). Positive transformants were selected for their ability to grow on Yeast‐Peptone‐Dextrose (YPD) plates containing zeocin at a final concentration of 100 μg/ml. *P. pastoris* X‐33 transformed with the empty vector pPICZαA was used as a negative control. Integration of the *Ba*EstB gene into the *P. pastoris* X‐33 genome was confirmed by colony PCR analysis using 5′ and 3′ AOX1 primers, following instructions of Easy Select *Pichia* Expression Kit (Life Technologies Invitrogen).

Positive transformants were grown in parallel on Minimal Dextrose (MD), and Minimal Methanol (MM) medium plates at 28°C for 48 hr to distinguish between Mut+ (Methanol utilization plus) and Muts (Methanol utilization slow) phenotypes. Five Mut+ colonies were selected. Strains wild type *P. pastoris* X‐33, *P. pastoris* X‐33 transformed with the empty vector (controls) and Mut+ transformed colonies designed as *Ba*EstB‐1–5 were inoculated into 15 ml Buffered Complex Medium containing Glycerol (BMGY) in 50 ml flasks and grown at 28°C on a rotary incubator (200–250 rpm) until the OD_(600)_ reached 2–6 (16–18 hr). Cells were harvested by centrifugation at 1,500*g* for 5 min and resuspended in 50 ml Buffered Complex Medium containing Methanol (BMMY) medium in 250 ml flasks at the same temperature and rpm. *Ba*EstB expression was induced adding methanol to a final concentration of 0.5% at 24 hr intervals during 4 days. Every day, 5 ml of culture was taken for monitoring esterase activity.

*Ba*EstB was purified from a 96‐hr culture supernatant using Nickel affinity chromatography. Briefly, the supernatant was recovered by centrifugation at 1,500*g* for 10 min and concentrated in a 30 kDa cut‐off amicon (GE healthcare). This concentrated preparation was loaded into the Ni‐column and eluted with a Tris‐HCl 10 mmol/L pH 7.5 and 0.2 mol/L imidazol solution. The purified enzyme was dialyzed and the protein was stored at 4°C until further use.

### 2.4 SDS‐PAGE and zymogram

Proteins from the crude extracts and from various purification steps were analyzed by sodium dodecyl sulphate polyacrylamide gel electrophoresis (SDS‐PAGE) according to the method of Laemmli (1970) using 4.5% stacking gel and 12.0% separating gel. The protein bands were stained with Coomassie brilliant blue R‐ 250. Page Ruler plus was used as molecular weight marker (ThermoScientific).

Esterase activity was assayed and visualized on zymograms, using 12.5% polyacrylamide gels (PAGE). Esterase activity staining was performed as described by Karpushova, Brümmer, Barth, Lange, & Schmid (2005). The gels were finally incubated for 5 min at room temperature in developing solution consisting of 3 mmol/L 2‐naphthyl acetate, 1 mmol/L Fast Garnet TR (Sigma) and 100 mmol/L sodium phosphate buffer, pH 7.5. The positive esterase activity was detected by the appearance of orange‐colored bands in the gels.

### 2.5 Esterase activity and substrate specificity determinations

Routinely, esterase activity was measured using 2‐naphthyl acetate as substrate (Soberanes Céspedes, Cruz, Vargas, & Vázquez, 2012). A 2‐naphthyl acetate stock solution (54 mmol/L, solution 1) was prepared in acetone. Solution 2 for substrate preparation was prepared as follows: 10 mg of Fast Garnet GBC sulfate salt was dissolved in 100 μl of 50 mmol/L potassium phosphate buffer (pH 7.01) and 0.1% Triton X‐100. Finally, a mixture of 10:0.01:0.05 (*v/v/v*) of potassium phosphate buffer (pH 7.01) 0.1% Triton X‐100/Solution 2/Solution 1, respectively was prepared. Briefly, 10 μl of diluted (1/1,000) pure esterase (1.3 mg of protein/ml) was added to the substrate solution (190 μl) plus 100 μl of 10 mmol/L Tris‐HCl buffer pH 7.0. The released 2‐naphtol was quantified by measuring the absorbance at 538 nm during 30 min. One unit of esterase activity is defined as the amount of enzyme releasing 1 μmol of 2‐naphtol per minute. The used molar absorption coefficient of 2‐naphtol was 23,598 M^−1^ cm^−1^.

The specificity of *Ba*EstB for the length of acyl chain was analyzed using *p*‐nitrophenyl acetate (*p*NPA, C_2_), *p*‐nitrophenyl butyrate (*p*NPB, C_4_), *p*‐nitrophenyl decanoate (*p*NPD, C_10_), and *p*‐nitrophenyl palmitate (*p*NPP, C_16_) as described previously (Selvin, Kennedy, Lejon, Kiran, & Dobson, 2012; Yan et al., 2014). One unit of esterase activity is defined as the amount of enzyme releasing 1 μmol of *p*‐nitrophenol (*p*NP) per minute. The used molar absorption coefficient of *p*NP at 410 nm was 17,800 M^−1^ cm^−1^.

The rhodamine B assay, specific for lipases, was conducted according to previous reports (Kouker & Jaeger, 1987). A stock solution with 2.5% (w/v) of olive oil was prepared and autoclaved. Rhodamine B (1 mg/ml) was dissolved in distilled water and filter sterilized (MILLEX^®^GV 0.2 μm). A solution containing 0.8% agar in buffer 10 mmol/L Tris‐HCl pH 7 was autoclaved and after cooling down to 60°C, 31.25 ml of the olive oil solution and 10 ml of the rhodamine B solution were added per L. This was poured in Petri dishes and when solidified, small wells were carved, filled with the different samples and incubated at 30°C for 16 hr, when the appearance of a fluorescent halo was detected under UV light (350 nm) in the positive control sample (a *Candida cylindracea* lipase, Sigma L1754).

The biochemical characterization of *Ba*EstB was performed using 2‐naphthyl acetate as substrate. All measurements were performed in triplicate.

### 2.6 Optimum temperature and stability of *Ba*EstB

Optimum temperature for the enzyme was investigated by measuring the esterase activity at different temperatures (10–60°C) with increments of 10°C. The thermal stability of the esterase was determined by measuring the residual activity of enzyme after a 60‐min preincubation employing the aforementioned temperature range with same increments. Specific activities were determined under the same condition mentioned above and using 2‐naphthyl acetate as substrate. All measurements were performed in triplicate.

### 2.7 pH optimum and pH stability of *Ba*EstB

Optimum pH of the enzyme was determined by varying the pH of the assay reaction mixture using the following buffers: 10 mmol/L Tris‐HCl (pH 3.0–7.0) or 10 mmol/L carbonate‐bicarbonate (pH 8.0–10.0). The stability of the esterase was determined after preincubating the enzyme in different buffer solutions (pH 3.0–10.0) for 60 min. The residual enzyme activity was then determined under the standard assay conditions. All measurements were performed in triplicate.

### 2.8 Kinetic parameters via isothermal titration calorimetry (ITC)

ITC experiments were performed on a Malvern ITC200 instrument by titrating 2‐naphthyl acetate into the enzyme suspension within the sample cell. Each single‐injection consisted in 38 μl of 2‐naphthyl acetate (170 μmol/L) and 37 pM of purified *Ba*EstB. All measurements were performed under the following conditions: temperature 30°C, stirring speed of 750 rpm, reference power of 8 μcal/s, and 90 min allowing the heat signal return to the baseline. The data were analyzed using Origin 7.0v software for *k*cat, *K*m, and ΔH determinations.

### 2.9 Effect of metal ions, NaCl, solvents, detergents, and EDTA on *Ba*EstB activity

In all the experiments described below, the specific activities were determined using 10 μl of diluted (1/1,000) pure enzyme (10.08 mg of protein/ml), 2‐naphthyl acetate as substrate and 10 mmol/L Tris‐HCl buffer (pH 7.0). All measurements were performed in triplicate.

### 2.10 Effect of metal ion on *Ba*EstB activity

The effect of several metal ions (Ba^2+^, Mg^2+^, Fe^2+^, Co^2+^, Ni^2+^, Mn^2+^, Zn^2+^, Cu^2+^, Ca^2+^ Ag^2+^, K^+^, Li^+^, Al^+^) on the *Ba*EstB activity was evaluated in the presence of 10 mmol/L of each metal ion and pH 7. Additionally, the *Ba*EstB stability was determined incubating the enzyme for 1 h at 30°C in Tris‐HCl buffer pH 7 in presence of the metal ions. The specific activity was measured and the residual activity was calculated.

### 2.11 Effect of NaCl on *Ba*EstB activity

*Ba*EstB activity was determined in different salinity conditions. Specific activity was measured by incubating the *Ba*EstB in Tris‐HCl buffer at 30°C with the addition of NaCl to final concentrations of 0.1, 0.25, 0.5, 1.0, and 2.0 mol/L in the reaction mixtures. The halostability (residual activity expressed in percentage) was also evaluated by incubating the *Ba*EstB 1 hr at 30°C in Tris‐HCl buffer in presence of 0.1, 0.25, 0.5, 1.0, and 2.0 mol/L NaCl.

### 2.12 Effect of solvents on *Ba*EstB activity

Specific activity *Ba*EstB in the presence of different solvents (30% *v/v* ethanol, methanol, acetone, isopropanol, 1‐butanol, chloroform, hexane, and DMSO) (Selvin et al., 2012) was measured by incubating *Ba*EstB in Tris‐HCl buffer at 30°C with the addition of the solvents in the reaction mixture.

### 2.13 Effect of detergents and EDTA on *Ba*EstB activity

The effect of several detergents (Tween 20, Tween 80, SDS, Triton X‐100, and CTAB) and EDTA on the *Ba*EstB activity was evaluated. Residual activity, expressed in percentage, was calculated by incubating the *Ba*EstB in Tris‐HCl buffer for 1 hr at 30°C in presence of 5% (*w/v*) of each compound (Yan et al., 2014).

### 2.14 Dynamic light scattering (DLS)

DLS experiments were conducted at 30°C using a Malvern Zetasizer Nano ZSP system. Translational diffusion coefficients (TDC) were obtained via measurements of the decay rates of scattered light and the autocorrelation curves. The hydrodynamic radius (H_R_) of the *Ba*EstB population was calculated from TDC on basis of the Stokes‐Einstein equation assuming a spherical geometry of the molecules. Samples were centrifuged at 12,000*g* for 5 min before the measurements. Two set of experiments, (1) *Ba*EstB without substrate and (2) *Ba*EstB in presence of substrate (2‐naphthyl acetate) were addressed; performing 12 scans for 10 s in both cases. DLS analysis of *Ba*EstB with 2‐naphthyl acetate was performed under the same conditions mentioned above considering its optimum pH and temperature. The data set replicates were analyzed using the DTS 5.10 software. Additionally, unfolding assays by temperature were performed in a range between 10 and 70°C. All measurements were performed in triplicate.

## 3 Results and Discussion

### 3.1 Nucleotide sequence of *Ba*EstB

A sequence with homology to the α/β hydrolase superfamily was identified from a cDNA library from *B. adusta* as described in materials and methods. The cDNA sequence consisted of a 969 nucleotide (nt) ORF (GenBank accession number KX580963) and its genomic counterpart comprised 1,133 nt which contained three introns of 57, 50, and 57 nt, respectively (FS1 supplementary material). The first twenty‐one hits of the PSI‐Blast showed approximately 40%–49% identity and 87%–93% coverage that were annotated as fungal hypothetical proteins or α/β hydrolases (FS2 supplementary material). The 253th hit was the first with an assigned function showing 26% identity and 60% coverage with an esterase from *Acinetobacter* sp. (GeneBank BAB68337.1). Therefore, PSI‐BLAST analysis suggests that this sequence belongs to the α/β hydrolase superfamily, however the majority of the proteins recovered from the PSI‐BLAST are hypothetical or uncharacterized proteins and further studies are needed to assign its function. As a first approach to unravel *Ba*EstB function, we decided to study its structure through bioinformatics analyses.

### 3.2 *Ba*EstB modeling

In the first attempt to model *Ba*EstB (I‐TASSER IDs: S243166 and S270524), the I‐TASSER platform selected as templates esterases/lipases from the PDB [4WY8 and 4WY5 (*Rhizomucor miehei*), 4ZRS (esterase from metagenome), 4N5I and 4PO3 (*Lactobacillus rhamnosis*), 2O7R (*Actinidia eriantha*), and 3WJ1 (*Ferroplasma* sp.)]. We obtained a first three‐dimensional model with TM‐score of 0.75 ± 0.11 and C‐score of 0.25. Being the PDB 4WY8 from a mucoral fungus and PDB 4ZRS the major templates, while other bacterial esterases/lipases and a plant carboxylesterase (PDB 2O7R from *Actinidia eriantha*) were also identified as templates by I‐TASSER. The identification of the top 10 structural analogs in PDB always revealed proteins belonging to the HSL family (EC 3.1.1.79) (Table 1), this analysis strongly suggests that *Ba*EstB is a member of this HSL family due to its structural similarity (Table 1, see TM‐score, Coverage and RMSD values).

**Table 1:** Top 10 identified structural analogs in PDB

Rank	PDB	TM	RMSD	Cov.	Source	Family
1	4WY8	0.867	2.01	0.929	*Rhizomucor miehei*	HSL
2	4WY5	0.844	2.27	0.916	*Rhizomucor miehei*	HSL
3	4OU4	0.839	2.03	0.904	*Pseudomonas putida*	HSL
4	1JKM	0.836	2.24	0.910	*Bacillus subtilis*	HSL
5	4J7A	0.832	2.22	0.904	Metagenomic library	HSL
6	4N5I	0.831	2.43	0.907	*Lactobacillus rhamnosis*	Not available
7	3ZWQ	0.822	2.27	0.894	*Pyrobaculum calidifontis*	HSL
8	1LZL	0.820	2.20	0.894	*Rhodococcus* sp.	HSL
9	1JJI	0.814	2.07	0.879	*Archaeoglobus fulgidus*	HSL
10	1QZ3	0.811	2.29	0.891	*Alicyclobacillus acidocaldarius*	HSL

Ranking of proteins is based on TM‐score of the structural alignment between the query structure and known structures in the PDB library.

TM: TM‐score.

RMSD is the RMSD between residues that are structurally aligned by TM‐align.

Cov.: Represents the coverage of the alignment by TM‐align and is equal to the number of structurally aligned residues divided by length of the query protein.

HSL: Hormone‐Sensitive Lipase.

**Figure 1:**
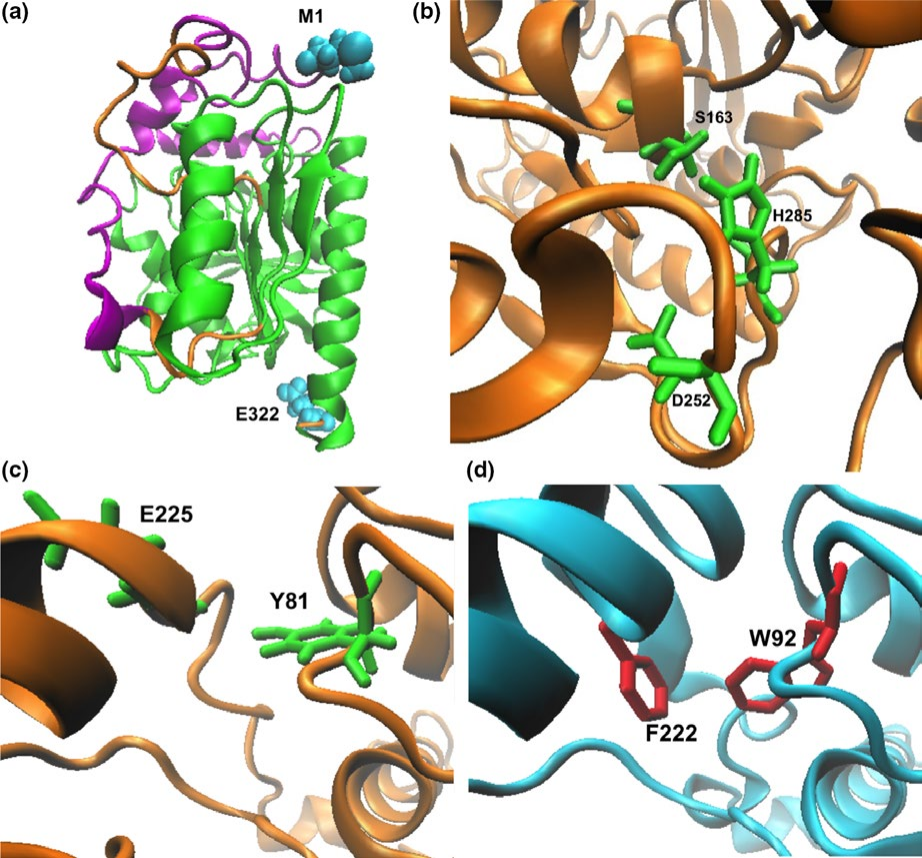
Structural analysis of *Ba*EstB. (a) Three‐dimensional model proposed for *Ba*EstB. The cap and catalytic domains are depicted in magenta and green, respectively; while the N‐terminal (M1) and C‐terminal (E322) are showed in cyan. (b) Catalytic triad of *Ba*EstB. S163, D252, and H285 are depicted in green. (c) and (d) Residues located in the center of the substrate‐binding pocket. (c) Y81 and E225 are depicted in green in *Ba*EstB, and (d) W92 and F222 are depicted in red in PDB 4WY8. All visualizations were performed in VMD

In order to obtain a more accurate three‐dimensional model, we submitted a new modeling round (I‐TASSER IDs: S248560 and S277038) using PDB 4ZRS and PDB 4WY8 as templates. The best model was obtained using PDB 4WY8 as template (Figure 1a) with a TM‐score of 0.83 ± 0.10 and a C‐score of 0.87. These score values confirm a high confidence in the quality of the model and additionally suggest the esterase/lipase activity of the *Ba*EstB. The model obeyed to the canonical α/β‐classical hydrolase architecture proposed by Ollis et al. (1992). During the second modeling, round I‐TASSER identified as templates esterases belonging to the HSL family: PDB 4WY8 and 4WY5 (*Rhizomucor miehei*) and 3ZWQ (*Pyrobaculum calidifontis*). Again, HSL were the closest structural neighbors. The results above suggest that *Ba*EstB belongs to HSL family.

Enzymes with lipolytic activity are classified into blocks C, L, H or X depending on their amino acid similarities and the presence of conserved motifs involved in the enzymatic catalysis (see ESTHER database) (Lenfant et al., 2013; Marchot & Chatonnet, 2012). Particularly, block H comprises two families: plant carboxylesterases and HSL, and its members are ubiquitous (see ESTHER database) (De Simone et al., 2004; Lenfant et al., 2013). According with this, it is not rare that PDB 2O7R was identified by I‐TASSER as a template during the first *Ba*EstB modeling round, probably being *Ba*EstB a member of block H. The HSL family is composed of esterases and lipases, and its members are widely represented in bacteria, plants, and animals (Tao et al., 2013). The HSL sequences identified in bacteria share high amino acid sequence similarity with the *LIPE* genes, which encode mammalian HSLs (Holm, 2003; Holm, Osterlund, Laurell, & Contreras, 2000). Two structural domains, cap and catalytic domains have been identified in HSL members, with the α/β‐classical hydrolase fold (Yang, Qin, et al., 2015). While several HSL have been structurally characterized in prokaryotic organisms (PDBs 2YH2, 1JJI, 3AIK, 1EVQ, 1JKM, etc.) (Dou et al., 2014; Ngo et al., 2013; Palm et al., 2011; Wei et al., 1999), only two HSL have been biochemically studied and crystallized in fungi (PDBs 4WY8 and 4WY5) (Yang, Qin, et al., 2015). 4WY8 and 4WY5 are the crystal structures of *Rm*EstB and *Rm*EstA, respectively, both esterases belonging to a member of the order Mucorales (Liu et al., 2013; Yan et al., 2014; Yang, Qin, et al., 2015). The structural alignments with the previous fungal HSL allowed identifying the cap (purple) and the catalytic domains (green) in *Ba*EstB (Figure 1a), as well as the catalytic triad, which was located in the respective canonical position. *Ba*EstB cap domain comprises residues 1–39 and 209–238, while the catalytic domain includes residues 40–193 and 244–316 and shows the distinctive molecular topology described for other HSL: an α/β‐hydrolase fold with a central β‐sheet of eight mostly parallel strands surrounded by α‐helices (Rozeboom, Godinho, Nardini, Quax, & Dijkstra, 2014; Yang, Qin, et al., 2015). The critical residues involved in the catalysis of *Ba*EstB were identified in the following positions: S163, D252, and H285 (Figure 17b), being the canonical catalytic residues for HSL family. The three‐dimensional superposition between PDB 4WY8 and *Ba*EstB showed that the average RMSDs for the catalytic triad (S163, D252, and H285 in *Ba*EstB vs. S164, D261, and H291 in PDB 4WY8) are 1.75, 5.28, and 4.68 Å, respectively.

Regarding the main structural characteristics of both, PDB 4WY8 and 4WY5, the critical residues located in the center of the substrate‐binding pocket were identified in *Ba*EstB also by structural alignments. *Ba*EstB showed a higher similarity to *Rm*EstB (4WY8) than to *Rm*EstA (4WY5; see below). While two aromatic residues (W92 and F222) are found in *Rm*EstB, an aromatic (Y81) and an acid residue (E225) are present in the centre of the *Ba*EstB′s substrate‐binding pocket (Figure 1c,d). The structural superposition allowed to calculate the average RMSDs for these residues between both proteins: 4.22 Å (W92 in PDB 4WY8 vs. Y81 in *Ba*EstB) and 3.27 Å (F222 in PDB 4WY8 vs. E225 in *Ba*EstB). It is possible that these residues could be implicated in the narrowing of substrate range.

**Figure 2:**
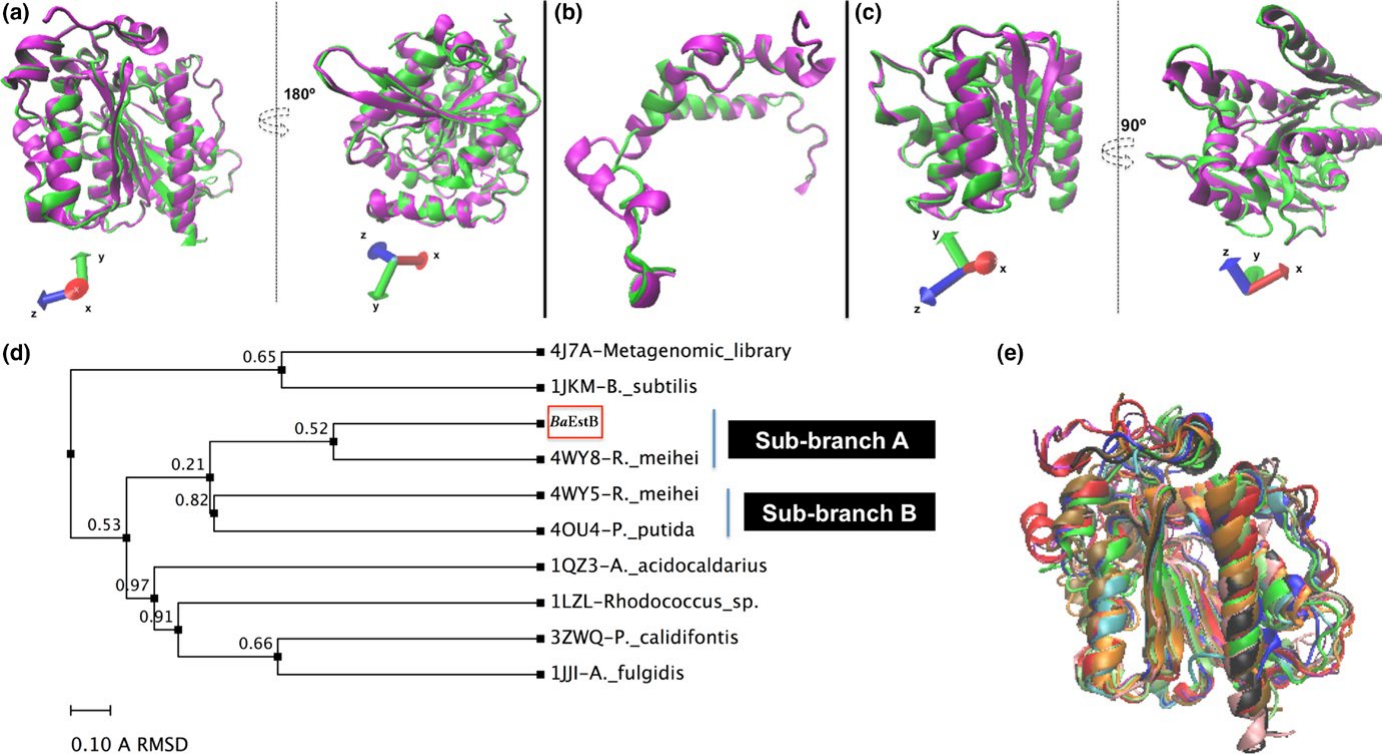
Superposition analysis between *Rm*EstB (PDB 4WY8) and *Ba*EstB. They are depicted in magenta and green, respectively. (a) Superpositioning of *Ba*EstB onto *Rm*EstB considering whole the sequence. (b) Superposition of cap domains. *Rm*EstB: residues 3–51 and 206–247, and *Ba*EstB: residues 1–39 and 209–238. (c) Superposition of catalytic domains. *Rm*EstB: residues 52–191 and 253–322, and *Ba*EstB: residues 40–193 and 244–322. (d) Structural‐based phylogenetic trees constructed according to the RMSD derived from the *Ba*EstB's structural comparison with its closest structural analogs. (d) *Ba*EstB′s superposition with its closest structural neighbors

A three‐dimensional superposition, considering the models derived from *Rm*EstB (PDB 4WY8) and *Ba*EstB, was prepared in VMD software previous a structural alignment of both models (Figure 2a). Values derived from the structural comparison such as TM‐score of 0.925, *Q*_H_‐score of 0.849, and an overall RMSD of 0.9304 Å for 322 corresponding Cα atoms, support a high structural homogeneity between both molecules (Figure 2a). The *Q*_H_‐score (metric value for structural alignment, *Q* = 1 when the proteins are identical), TM‐score, and RMSD values are used to determine how good an alignment is between two proteins, and at the same time how similar they are structurally. The main differences of the molecular architectures were found in the loop regions, while the cap domain and the core of the enzymes exhibited a high structural superposition, showing a tertiary structure similarity (Figure 2b,c). These results confirm that *Ba*EstB has the HSL canonical topology. Furthermore, superpositioning of *Ba*EstB onto *Rm*EstB revealed better overlay than the structural comparison between *Rm*EstA and *Rm*EstB according to the overall RMSD obtained for both analysis: 0.9304 versus 1.5117 Å, respectively. In this structural comparison, the *Q*_H_ value was also best for the *Ba*EstB‐*Rm*EstB (0.849) superposition than that for *Rm*EstA‐*Rm*EstB overlap (0.766). These data are interesting since *R. miehei* (Glomeromycota, subphylum Mucoromycotina) and *B. adusta* (Basidiomycota) are not phylogenetically close.

A structure‐based phylogenetic tree constructed confirmed the results above according with the RMSD values derived from the *Ba*EstB's structural comparison with its closest structural analogs (recovered by I‐TASSER during our second modeling round) deposited in PDB (Figure 2d). This phylogenetic analysis showed that while *Ba*EstB is grouped directly with *Rm*EstB (PDB 4WY8) in the sub‐branch A, *Rm*EstA and *Rm*EstB are resolved in different sub‐branches (A and B), being *Rm*EstA (PDB 4WY5) directly related with a *Pseudomonas putida*′s esterase (PDB 4OU4) grouped as sub‐branch B (Figure 2d). These relations are supported by the average RMSD values obtained for both structural comparisons, and demonstrate that *Rm*EstB is the characterized HSL microbial protein more phylogenetically related with *Ba*EstB. Additionally, *Ba*EstB was structurally overlaid with 10 PDB, recovered by I‐TASSER, obtaining high structural similarity (Figure 2e). Superpositions *Q*_H_ values were between 0.6174 for PDB 1JKM and 0.849 for *Rm*EstB as mentioned earlier, while RMSD values were up to 2.4934 Å. RMSD values of 2.4370, 2.4934, 2.1310, and 2.0931 Å were obtained for the topological comparison with PDB 4J7A, 1JKM, 1JJI, and 3ZWQ, respectively. These results are coherent with the distant phylogenetic relationship shown in Figure 2d. Overall, these data demonstrate that the basic architecture of HSL proteins is highly conserved in different microorganisms.

### 3.3 Structural alignment analysis

Table 1 shows the nine crystallized HSL deposited in PDB that were used to perform the structural alignment, which were identified as its closest structural neighbors. The best alignment (17.10% of identity) is shown with PDB 3ZWQ (*P. calidifontis*), while the lowest percentage identity (11.87%) was observed with a bacterial HSL homolog isolated from a metagenomic library (PDB 4J7A). The percentage identities between *Ba*EstB vs. *Rm*EstA and *Rm*EstB were 15.63% and 16.91%, respectively (data not shown). Although the 10 sequences have low identity, they display a conserved molecular architecture. It has been reported in previous studies (Yang, Qin, et al., 2015) that cap domains are the worst alignment regions, however they share their tertiary structure as Figure 2b shows. Particularly, *Ba*EstB does not exhibit sequence similarity in the cap domain with other HSL, not even with the fungal HSL (*Rm*EstA and *Rm*EstB).

**Figure 3:**
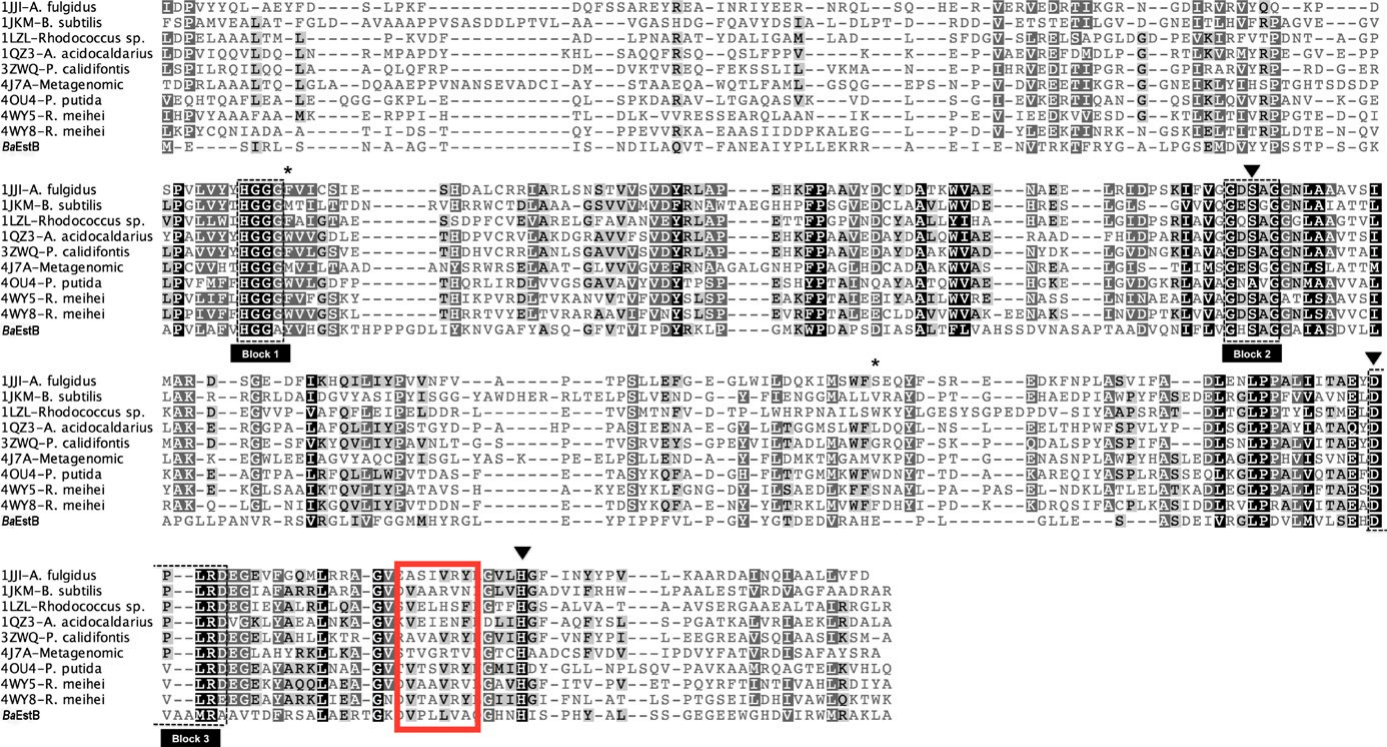
Structural alignment of *Ba*EstB with HSL deposited in PDB: 4WY8, 4WY5, 4OU4, 1JKM, 4J7A, 3ZWQ, 1LZL, 1JJi and 1QZ3. The catalytic triad (Ser‐His‐Asp) is indicated with black triangles, while the amino acids located in the center of the substrate‐biding pocket are marked with black asterisks. The alignment shows the typical domains in HSL esterases. Identical residues are shaded in black, and conserved residues are shaded in gray. The conserved catalytic motif is underlined. The three signature motifs for HSL proteins named Block 1, 2, and 3 are indicated as black dotted boxes. The putative catalytic nucleophile and acid/base are identified by a black filled arrow

From the alignment of the analyzed sequences, the HSL canonical catalytic triad (Ser‐His‐Asp) was located (Figure 3). In *Ba*EstB, Ser163 is the nucleophile, His285 is the proton acceptor/donor, and Asp252 is the amino acid stabilizing His285. Additionally, the three signature motifs for HSL proteins were identified in *Ba*EstB, and they were named as Block 1, 2, and 3 (Figure 3). The conserved tetrapeptide motif His‐Gly‐Gly‐Gly (Block 1 in Figure 3) involved in the oxyanion cavity formation was identified in the aligned sequences and it was located upstream of the active site. *Ba*EstB sequence shows a conservative substitution in this motif (^77^His‐Gly‐Gly‐Ala ^80^), being this structural characteristic never reported for these proteins. It has been demonstrated that an Ala residue downstream of this signature motif is necessary to create the oxyanion cavity (Ngo et al., 2013; Yang, Qin, et al., 2015). We could find it in position 164 of *Ba*EstB, being this amino acid extensively conserved in these sequences including the *Rhizomucor miehei*′s HLS (Ala163 and Ala165 in *Rm*Est A and *Rm*EstB, respectively); being Gly78, Gly79, and Ala164 the three amino acids involved in the oxyanion cavity formation in *Ba*EstB.

On the other hand, the nucleophilic Ser163 is located in the characteristic ^160^Gly‐*X*‐Ser‐*X*‐Gly^164^ pentapeptide sequence (Block 2 in Figure 3). Some authors extend this block three more residues (Ngo et al., 2013), however these positions are less conserved in the block although it can define a consensus extended motif Gly‐X‐Ser‐X‐Gly‐Gly‐Asn‐Leu. Gly and Leu being the most conserved and that are present in *Ba*EstB: ^160^Gly‐X‐Ser‐X‐Gly‐Gly‐Ala‐Ile ^167^. A conservative change (Leu for Ile) and a nonconservative change (Asn for Ala) are present in *Ba*EstB, while similar substitutions are found in the other sequences. A third conserved block Asp‐Pro‐*X*‐*X*‐Asp (Block 3 in Figure 3) involved in catalysis is common in HSL. *Ba*EstB Block 3 is composed by ^252^Asp‐Val‐**Ala**‐**Ala**‐X‐X‐Ala^258^. The insertion of two Ala residues, is a distinctive structural characteristic for *Ba*EstB since in the other sequences it is not present. Moreover, in *Ba*EstB and in both *R. miehei*′s HSL, a Pro residue is changed by Val. This substitution may be typical of fungi, since it is rarely found in bacteria (i.e., PDB 4OU4 of *P. putida*). Additionally, a nonconservative change is found in *Ba*EstB, where an Asp is replaced by Ala. However, the positions *X‐X* in the Asp‐Pro‐*X*‐*X*‐Asp motif is conserved (L and R) among all the sequences studied except in *Ba*EstB (where we found M and R). Given this analysis, we cannot consider that the third block is conserved in *Ba*EstB.

### 3.4 Sequence phylogenetic analysis

**Figure 4:**
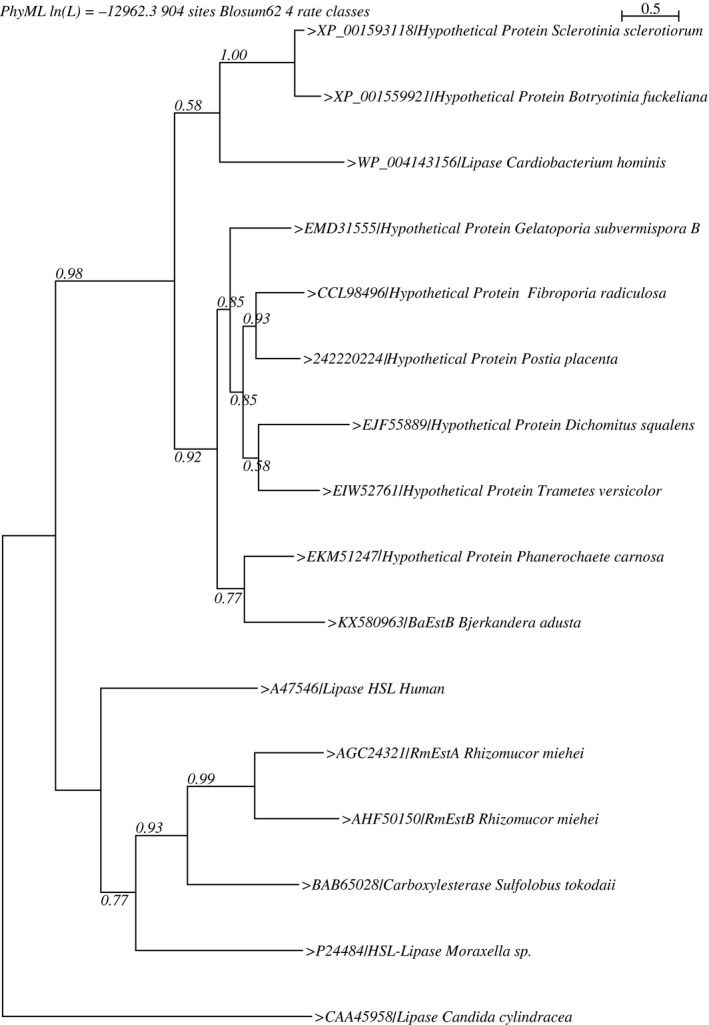
Phylogenetic reconstruction of *Ba*EstB. The Maximum Likelihood method was used to estimate the phylogenetic tree; the branch support was assessed using the *ALTr* algorithm and the Jones‐Thornton‐Taylor (JTT) model was used to estimate distances for amino acids

The phylogenetic analysis based on the amino acid sequence showed that *Ba*EstB is not grouped directly with any HSL member considered in the phylogeny reconstruction (Figure 4). This result is not surprising because *Ba*EstB is a fungal protein with some distinctive structural characteristics as mentioned above. This relation could be supported by the low similarity coefficients observed in the multiple structural alignments. *Ba*EstB is not directly related with *Rm*EstA and *Rm*EstB, which are also fungal proteins but from another distant Phylum (Glomeromycota, subphylum Mucoromycotina), which form a clade with bacterial HSL (Figure 4). The phylogeny suggests that these proteins presumably have a common ancestor since prokaryotic and fungal HSL share different clades. On the other hand, *Ba*EstB groups with very good support values within a clade of hypothetical proteins all from Basidiomycetes. Taken together with the Blast results in which the first 21 hits showed identities of approximately 40%–49% (vs. 16.91% identity with *Rm*EstB from *R. miehei*) and 87%–93% coverage (FS2 supplementary material), these results may suggest that the proteins in the first hits of the Blast could be HSL esterases in Basidiomycetes from the class Agaricomycetes.

### 3.5 Expression and purification of *Ba*EstB in *P. pastoris*

**Figure 5:**
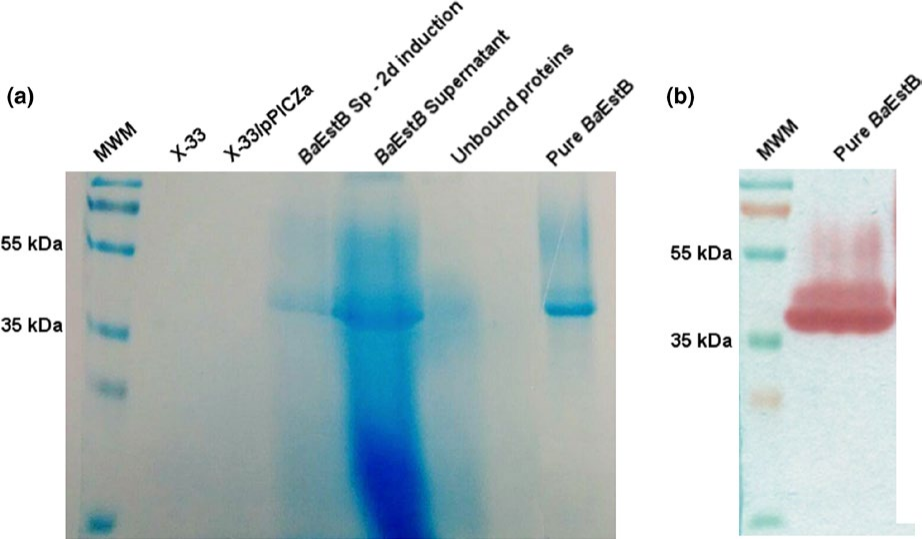
SDS‐PAGE and zymogram analysis of the purified esterase from *B. adusta* (*Ba*EstB). (a) Purification steps of *Ba*EstB and (b) zymogram analysis of esterase on SDS‐polyacrylamide gel using 2‐naphthyl acetate as substrate

Oligonucleotides were designed to amplify the coding sequence of the putative esterase to be inserted in pJET, an intermediate vector. The cDNA was then subcloned in the expression vector pPICZαA to be transformed in *P. pastoris* as has been described in material and methods section. Several independent colonies were induced with 0.5% methanol in BMMY medium and a qualitative assay using 2‐naphthyl acetate was performed to select a colony that produced the best activity. *P. pastoris* wild type strain X‐33 and a strain X‐33 transformed with the empty vector showed no esterase activity. Three out of six colonies were selected to perform a quantitative assay from culture supernatants. Strain *Ba*EstB5 was selected for further experiments. From this clone, *Ba*EstB was purified through nickel affinity chromatography with 2.9‐fold purification with recovery of 17% and specific activity of 31.58 U/mg using 2‐naphthyl acetate (Table 2). The purified enzyme showed a single protein band both on SDS‐PAGE and in a zymogram with an estimated molecular mass of 38.3 kDa (Figure 5a,b). The purified enzyme was used for its biochemical characterization.

**Table 2:** Results of the HSL purification from the heterologous expression system *P. pastoris*

	Volumetric activity (U/L)	Volume (ml)	Total units	Total protein	Specific activity (U/mg)	Purified fraction	Recuperation (%)
Supernatant‐*Ba*EstB	39.9	43	1.71	149.03	11	1	100
Pure *Ba*EstB	193.15	1.5	0.29	15.2	31.58	2.9	17

### 3.6 Biochemical characterization of *Ba*EstB

#### 3.6.1 Substrate specificity

Since the α/β hydrolase family comprises both esterases and lipases, we performed a specific assay for lipases using rhodamine B together with commercial olive oil and monitoring the appearance of a fluorescent halo in ultra thin agar plates at *λ* 350 nm (Kouker & Jaeger, 1987). Concentrated supernatants of the wild type (wt) and a strain transformed with the empty vector or the purified *Ba*EstB preparation did not show a fluorescent halo, contrary to a *Candida* lipase that was used as a positive control, showing that *Ba*EstB has no lipase activity (data not shown).

In order to evaluate the substrate specificity of the enzyme, different *ρ*‐NP esters with acyl chain lengths from C2 (*ρ*‐NP acetate) to C16 (*ρ*‐NP palmitate) were tested. Table 3 shows that enzyme activity declined along with longer chain‐length, reaching 1.78 ± 0.33 U/mg with *ρ*‐NP acetate (C2) and 0.99 ± 0.12 U/mg with *ρ‐*NP butyrate (C4). Maximal hydrolytic activity was obtained against 2‐naphthyl acetate with 31.58 ± 0.1 U/mg (C2) and no activity was detected against *ρ‐*NP esters with acyl chain lengths longer than C6. Together, these results indicated that the enzyme was an esterase with preference for short‐chain esters. It is worth to note that the substrate specificity could be influenced by the size of the other moiety of the molecule, since 2‐naphthyl acetate has two rings while *ρ*‐NP esters have only one. If we compare the activity obtained with *ρ*‐NP acetate versus that with 2‐naphthyl acetate, an almost 18‐fold difference is noted although the acyl chain is the same (acetate). This notion is supported by the fact that other esterases such as acetyl xylan esterases from family CE4, show a low activity on *ρ*‐NP esters while they are very active on xylan, which is a polymer of xylopyranosyl residues. The difference on this activity is proposed to be due to the architecture of the neighboring sugars that allow the enzyme to better position itself in order to hydrolyze the acetate group (see Figure 12 in Biely, 2012) (Biely, 2012). As mentioned above, in mammals, HSL have bulky substrates such as cholesteryl esters. Taken together, these ideas suggest that *Ba*EstB could be involved in unesterifying ergosterol esters, which have been detected by Yuan, Kuang, Wang, and Liu (2008) in different fungal tissues.

**Table 3:** Specific activity for of different substrates

Substrate	Specific activity (μmol/mg)
2‐Naphthyl Acetate	31.58 ± 0.1
*ρ*‐NP Acetate	1.78 ± 0.329
*ρ*‐NP Butyrate	0.98 ± 0.115
*ρ*‐NP Decanoate	0
*ρ*‐NP Palmitate	0

#### 3.6.2 Effect of temperature and pH on *Ba*EstB activity

**Figure 6:**
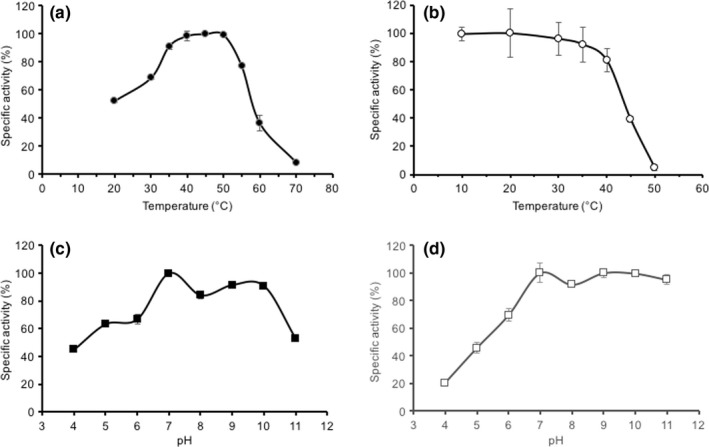
Effect of temperature and pH on the activity and stability of recombinant *Ba*EstB. (a) Optimal temperature of *Ba*EstB activity. (b) *Ba*EstB thermal stability. (c) pH optimum for *Ba*EstB activity and (d) pH stability for *Ba*EstB

Optimal temperature and pH were determined for *Ba*EstB over a temperature range from 10°C to 70°C and pH 4–11. This enzyme showed an optimal activity at 45°C (31.58 ± 0.1 U/mg), whereas at 30°C and 50°C still conserves 69% and 99% of activity, respectively. At lower temperatures, 20°C, it only shows 52% of the optimal activity and at 70°C it is almost inactivated. (Figure 6a). Thermostability of the enzyme was also determined. After incubation at different temperatures, we could determine that *Ba*EstB conserves almost 81% of its activity after incubation at 40°C for 1 hr. Incubation at 10°C to 30°C showed around 97% of its activity, whereas incubation at 50°C abolished the activity to 5% (Figure 6b). HSL counterparts in *Rhizomucor* show similar optimal temperatures between 45 and 50°C (Liu et al., 2013; Yan et al., 2014), while other esterases from metagenomic libraries have their optimal temperatures around 30°C (Jeon et al., 2012; Li et al., 2014, 2015). Considering the temperature, *Ba*EstB is a mesophilic HSL esterase, compared with a thermostable esterase from *Thermoanaerobacter tengcongensis* with optimal temperature at 70°C (Rao et al., 2011).

The purified *Ba*EstB showed relatively high activity under alkaline conditions, and exhibited optimum activity at pH 7.0 in 50 mmol/L Tris–HCl (100%). The activity diminished only to 91% at pH 10; whereas at pH 11, the activity decreased to 53%. At the acidic pH 4, the enzyme has only 45% of activity (Figure 6c). We also determined the stability of the enzyme at different pH by incubating it in different buffers for 1 hr and then determining the activity. The most stable condition was found to be pH 7.0 showing 100% of activity, whereas at pH 6 the enzyme retained 69% of the activity. At more basic pH (from 8 to 10), the enzyme showed to be more stable since retained more than 90% of activity. However, at acidic pH 4, only around 20% residual activity was found (Figure 6d). As it was reported by Liu et al. (2013) for the *R. miehei* HSL enzymes, *Ba*EstB also shows better activity in pHs around neutrality although it retains 50% of its activity even at pH 11. Compared with other HSL esterases, *Ba*EstB retains its activity in a wider alkaline pH range. *Ba*EstB optimal pH of 7.0 is lower than other esterases like that of an oil‐degrading bacterium (pH 8.5) (Mizuguchi et al., 1999), *Pseudoalteromonas* sp (pH 8) (Cieśliński et al., 2007) or *Thermoanaerobacter tengcongensi* (pH 9.5) (Rao et al., 2011), but higher than an esterase from the basidiomycete *Pleurotus sapidus* (pH 6) (Linke et al., 2013).

#### 3.6.3 kcat, Km, and ∆H determinations via ITC

**Figure 7:**
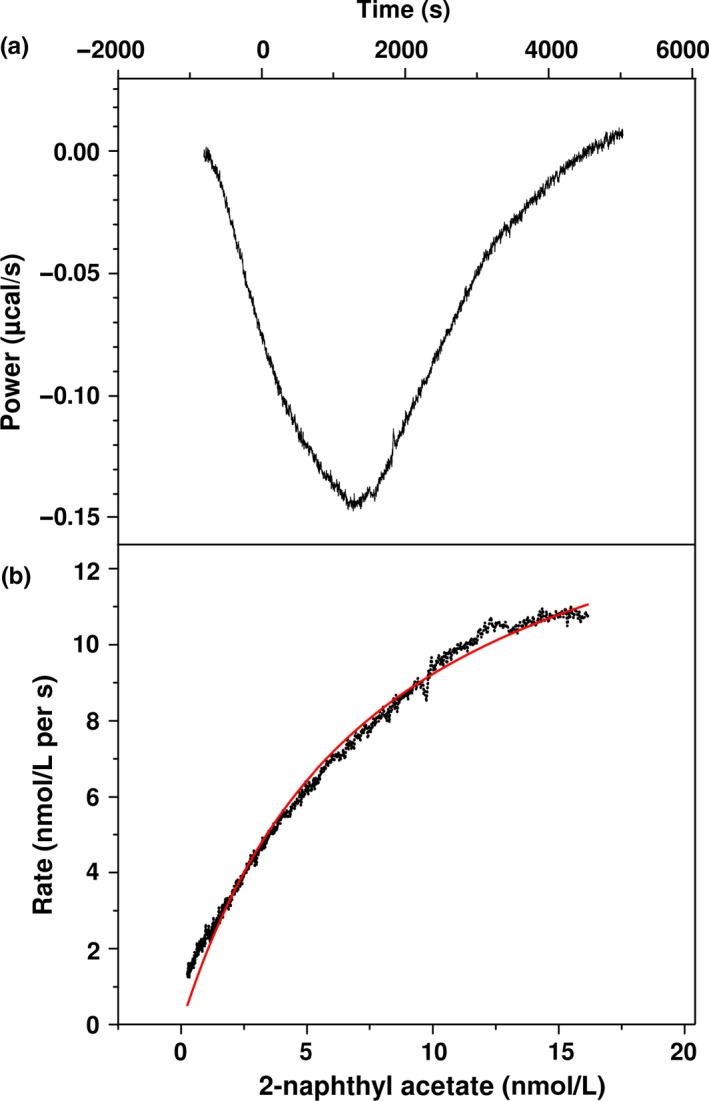
ITC analysis. (a) Curve plotting the “heat” values (μcal/s) from the enzymatic reaction during 5,400 s. (b) Curve obtained from product formation velocity (nmol/L per s) versus 2‐naphthyl acetate concentration (nmol/L)

The kinetic parameters of *Ba*EstB were determined via ITC using its optimum pH and temperature. *Ba*EstB′s kinetic properties were obtained plotting the “heat” values from the enzymatic reaction during 90 min (Figure 7a). The curve obtained from product formation velocity versus substrate concentration showed a high‐quality adjustment via Michaellis–Menten equation (*χ* ^2^ = 1.153 × 10^−13^) (Figure 7b), which is integrated in the used software. *Ba*EstB showed a *k*cat_A_ of 5.31 × 10^3^ ± 20 s^−1^ and *K*m_A_ of 7.68 ± 0.62 μmol/L. The 2‐naphthyl acetate hydrolysis is enthalpically favored by *Ba*EstB (∆*H* = −6.54 × 10^4^ cal/mol).

#### 3.6.4 Effects of metals, solvents, and detergents on *Ba*EstB activity

The effects of various chemicals on the enzymatic activity were evaluated by measuring initial and residual activity after incubation (Table 4). The initial value is referred to that measured in a control sample without the chemical, taken as 100% activity. Metal ions that slightly affected activity were Li^+^, K^+^, and Mg^2+^. The *Ba*EstB activity was strongly inhibited by Cu^2+^ (0.15%), Al^+^ (0.68%), Fe^2+^ (0.78%), and Ag^2+^ (7%), and moderately inhibited by Mg^2+^ (74%), Li^+^ (81.3%), and K^+^ (75%). After an hour of incubation in the presence of these metals, the stability of the esterase is low. Also *Ba*EstB shows slight halotolerance, at 0.1 mol/L NaCl the enzyme retained almost all its activity (91%) and it only diminished to 30% in the presence of 1 mol/L or 2 mol/L NaCl indicating a slight tolerance to salinity. In contrast, an esterase isolated from a metagenomic library has a high halotolerance showing more activity in presence of salt (Selvin et al., 2012).

**Table 4:** Specific activity and residual activity in (%) of *Ba*EstB with different divalent and monovalent ions and NaCl concentrations

Metal ions	Specific activity	Residual activity
No ions/No NaCl	100 ± 1.63	44.57 ± 1.78
Ba^2+^	40.57 ± 4.21	41.00 ± 7.07
Mg^2+^	73.64 ± 1.02	25.61 ± 3.11
Ca^2+^	39.81 ± 3.48	47.28 ± 5.50
Mn^2+^	28.82 ± 3.72	17.31 ± 4.84
Fe^2+^	1.78 ± 0.35	0.76 ± 0.21
Co^2+^	54.36 ± 3.97	12.13 ± 0.83
Ni^2+^	15.53 ± 2.92	0.29 ± 0
Cu^2+^	0.15 ± 0.03	0.11 ± 0.0001
Zn^2+^	18.72 ± 2.21	1.53 ± 0.24
Ag^2+^	6.88 ± 0.91	4.19 ± 0.88
K^+^	74.64 ± 8.73	33.8 ± 3.22
Li^+^	81.13 ± 1.54	43.06 ± 1.16
Al^+^	0.68 ± 0.16	0.13 ± 0.03
NaCl concentration		
0.1 mol/L	91.29 ± 9.53	61.72 ± 0.78
0.25 mol/L	82.12 ± 13.19	43.01 ± 2.38
0.5 mol/L	69.42 ± 10.11	43.00 ± 0.80
1.0 mol/L	34.85 ± 2.58	39.71 ± 1.04
2.0 mol/L	30.99 ± 4.36	39.43 ± 1.74

Values represent the means of three replicates ± standard error.

The enzyme activity of *Ba*EstB was significantly inactivated in the presence of methanol, isopropanol, ethanol, acetone, DMSO, and hexane. In the presence of 1‐butanol and chloroform, the reduction was not as severe, showing 50% and 82%, respectively (Table 5). However, in all the cases, the residual activity was low. Furthermore, the enzyme's activity was significantly abolished in the presence of different detergents such as Tween 20, Tween 80, Triton X‐100, SDS and CTAB, as well as a chelant agent, EDTA (Table 6).

**Table 5:** Specific activity and residual activity in (%) of the *Ba*EstB in the presence of solvents

Solvent	Specific activity	Residual activity
Not solvent	100 ± 1.63	44.57 ± 1.78
Ethanol	22.00 ± 0.44	0.90 ± 0.14
Methanol	6.18 ± 1.23	8.45 ± 1.01
Isopropanol	19.49 ± 0.95	0.73 ± 0.27
Butanol	49.48 ± 4.14	0.35 ± 0.10
Acetone	7.33 ± 0.37	0.49 ± 0.09
Acetonitrile	3.55 ± 0.74	0.29 ± 0.41
Chloroform	82.79 ± 6.04	16.98 ± 3.09
Hexane	40.14 ± 4.55	64.77 ± 4.32
DMSO	13.10 ± 1.55	36.11 ± 3.25

Values represent the means of three replicates ± standard error.

**Table 6:** Specific activity and residual activity in (%) of *Ba*EstB in the presence of detergents

Detergent	Specific activity	Residual activity
Not detergent	100 ± 1.63	44.57 ± 1.78
Tween 20	0.27 ± 0.08	0.74 ± 0.34
Tween 80	0.69 ± 0.17	1.55 ± 0.54
Triton X‐100	16.14 ± 3.79	43.47 ± 0.96
SDS	0.33 ± 0.06	0.49 ± 0.34
CTAB	2.22 ± 0.37	1.10 ± 0.44
EDTA	29.91 ± 7.37	51.03 ± 4.67

#### 3.6.5 DLS analysis

**Figure 8:**
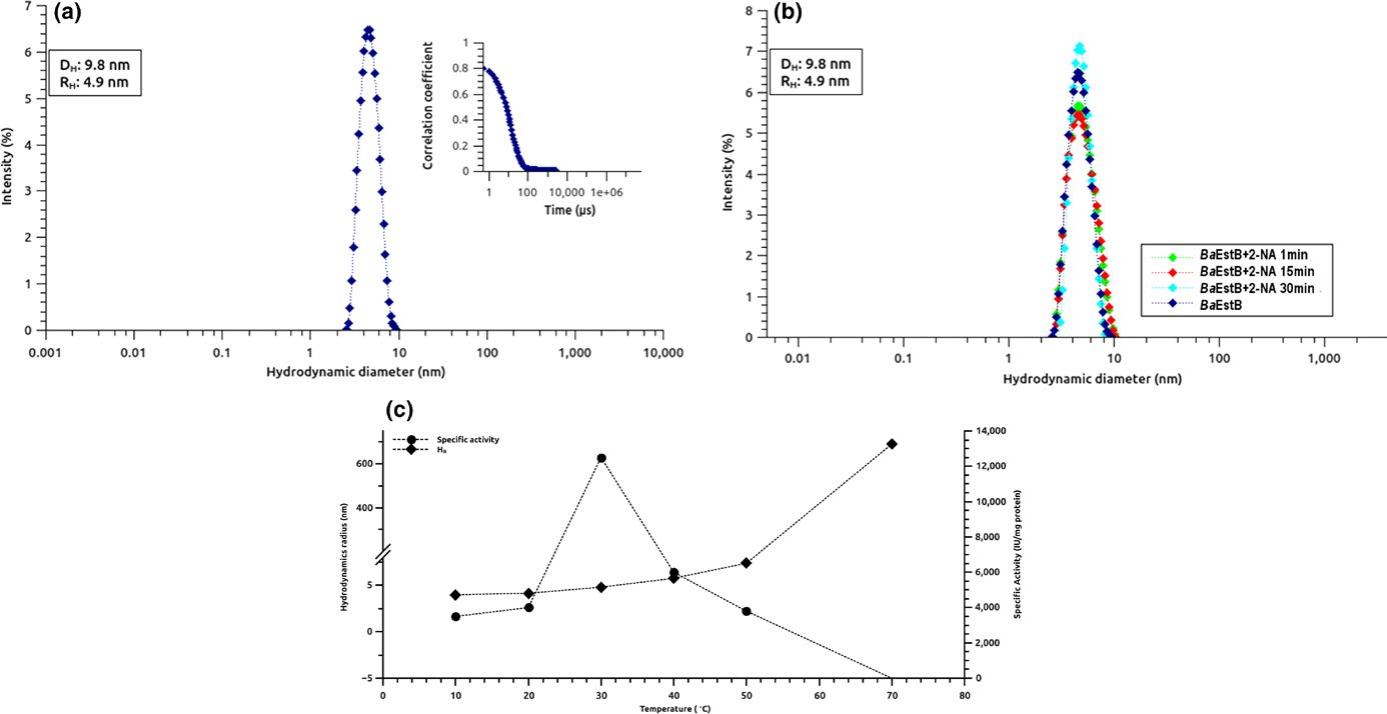
DLS analysis. (a) Hydrodynamic diameter determination of *Ba*EstB population without substrate. (b) Hydrodynamic diameter determination of *Ba*EstB population in presence of 2‐naphthyl acetate. (c) Relationship between hydrodynamic radius and temperature

Figure 8a depicts the light scattering intensity (%) versus the hydrodynamic diameter (nm), showing a single *Ba*EstB population with a *H*_R_ of 4.9 nm. This value suggests a dimeric state for *Ba*EstB according to the expected *H*_R_ (5.4 nm) for a protein of 38.3 KDa (monomeric state). The correlation curve (Figure 8a, upper) supports the high quality of DLS analysis. A second experiment with *Ba*EstB preparations in the presence of 2‐naphthyl acetate was performed in order to demonstrate any effect of the substrate on the oligomeric state of the enzyme. Figure 8b suggests that the dimeric conformation of *Ba*EstB is maintained during its interaction with the substrate (the same *H*_R_ value was obtained).

Figure 8c shows the relationship between hydrodynamic radius and temperature. Hydrodynamic radius values increase while temperature is higher, indicating the unfolding of *Ba*EstB. The hydrodynamic radius at 70°C suggests that *Ba*EstB is totally unfolding and it is in correspondence with its specific activity at this temperature (0 IU/mg protein). Other HSL enzymes, as PDB 4J7A isolated from a metagenome (Ngo et al., 2013) and PDB 4WY5 from *R. miehei* (Yang, Qin, et al., 2015), have been also reported as dimers, the latter forming a tetrameric structure through hydrogen bonding; supporting our results that show that *Ba*EstB is constituted as a dimer.

## Conclusions

In this work, we report for the first time a HSL from a Basidiomycete fungus. The genomic sequence contains three small introns which is not unusual for Basidiomycetes. Through three‐dimensional modeling studies and phylogenetic analysis, we conclude that *Ba*EstB is an ortholog of the previously described *Rm*EstB HSL of *R. miehei*. Traditional phylogenetic analysis together with Blastx results, suggests that a number of fungal hypothetical proteins could belong to the HSL family.

## Conflict of Interest

The authors declare that they have no conflicts of interest with the contents of this article.
