# Supplementary material for: The first description of a hormone‐sensitive lipase from a basidiomycete: Structural insights and biochemical characterization revealed Bjerkandera adusta BaEstB as a novel esterase
Source: Microbiologyopen. 2017 Mar 1;6(4):e00463. doi: 10.1002/mbo3.463 (PMC5552909; doi:10.1002/mbo3.463)
Supplement: Supplementary file 1 [file MBO3-6-na-s001.pdf]

Supplementary figure S1

```
Geno ATGGAATCTATCCGTCTGTCCAACGCTGCAGGCACGATCTCGAATGACATCCTGGCC
cDNA ATGGAATCTATCCGTCTGTCCAACGCTGCAGGCACGATCTCGAATGACATCCTGGCC
     *********************************************************
Geno CAGGTCACTTTTGGTATGTCGGTTGCCTCAGCCTGAGAGTCAACACAGGCTCACTAAATT
cDNA CAGGTCACTTTTG-----------------------------------------------
     *************
Geno TCATGGACAGCGAACGAAGCTATCTACCCTCTCTTAGAAAAGCGACGCGCCGAAATTGAA
cDNA ----------CGAACGAAGCTATCTACCCTCTCTTAGAAAAGCGACGCGCCGAAATTGAA
               **************************************************
Genó AATGTGACTCGCAAGACGTTTCGGTACGGTGCTCTCCCCGGCAGCGAGGTGTGTCTCGTC
cDNA AATGTGACTCGCAAGACGTTTCGGTACGGTGCTCTCCCCGGCAGCGAG------------
     ************************************************
Genó CAGGCTGTTGCTGCGAACGTAGTGCTCAGGAAATACAGATGGACGTTTACTACCCTTCTT
cDNA --------------------------------------ATGGACGTTTACTACCCTTCTT
                                           **********************
Geno CCACACCGTCCGGAAAGGCCCCTGTCCTAGCTTTTGTCCACGGCGGAGCCTATGTACACG
cDNA CCACACCGTCCGGAAAGGCCCCTGTCCTAGCTTTTGTCCACGGCGGAGCCTATGTACACG
     ************************************************************
Geno GATCGAAAACCCACCCTCCTCCTGGTGACCTGATCTACAAGAACGTCGGGGCATTCTACG
cDNA GATCGAAAACCCACCCTCCTCCTGGTGACCTGATCTACAAGAACGTCGGGGCATTCTACG
     ************************************************************
Geno CCTCACAAGGGTACGCACTTCGATCGTAGCCACTGCCGGCTTTCTCCGCTCAACTAATAA
cDNA CCTCACAAGG--------------------------------------------------
     **********
Geno AACGTAGCTTCGTCACCGTCATACCCGACTACCGCAAGCTCCCAGGCATGAAATGGCCCG
cDNA -------CTTCGTCACCGTCATACCCGACTACCGCAAGCTCCCAGGCATGAAATGGCCCG
            ***************************************************** !!
```
