# Supplementary figures and images for: The first description of a hormone‐sensitive lipase from a basidiomycete: Structural insights and biochemical characterization revealed Bjerkandera adusta BaEstB as a novel esterase
Source: Microbiologyopen. 2017 Mar 1;6(4):e00463. doi: 10.1002/mbo3.463 (PMC5552909; doi:10.1002/mbo3.463)

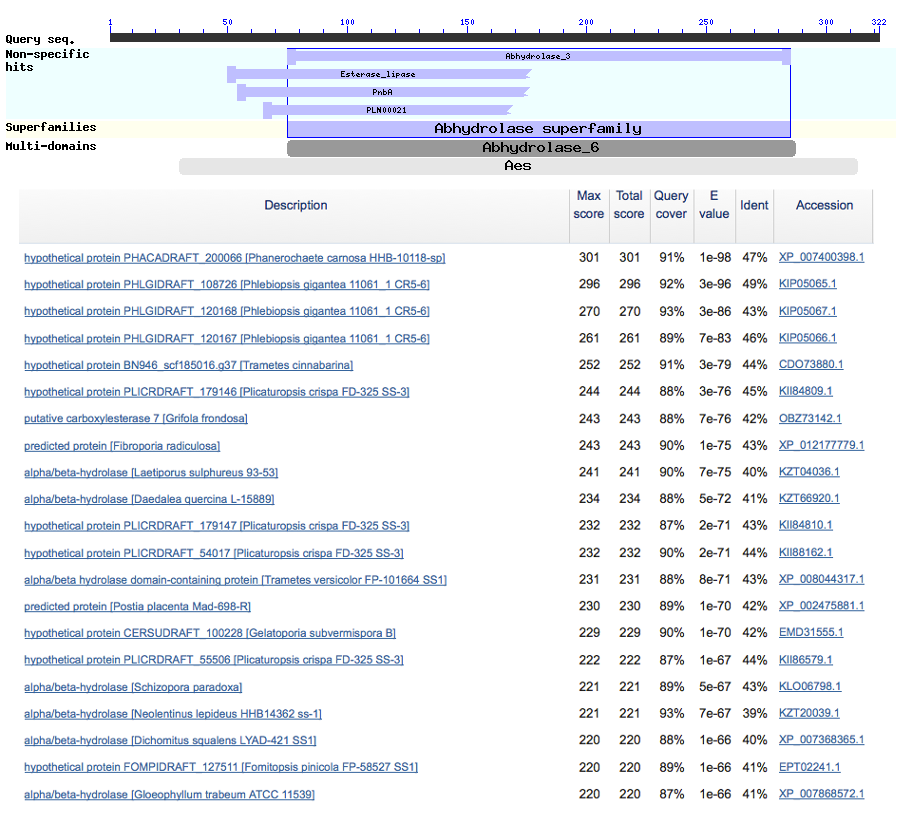

Supplement: Supplementary file 2 [file MBO3-6-na-s002.tiff]
