# Supplementary material for: The first description of a hormone‐sensitive lipase from a basidiomycete: Structural insights and biochemical characterization revealed Bjerkandera adusta BaEstB as a novel esterase
Source: Microbiologyopen. 2017 Mar 1;6(4):e00463. doi: 10.1002/mbo3.463 (PMC5552909; doi:10.1002/mbo3.463)
Supplement: Supplementary file 3 [file MBO3-6-na-s003.doc]

Table S1. Matrix of identity percentages derived from the structural alignment.

|  | **1JJI** | **1JKM** | **1LZL** | **1QZ3** | **3ZWQ** | **4J7A** | **4OU4** | **4WY5** | **4WY8** | **BaEstB** |
| --- | --- | --- | --- | --- | --- | --- | --- | --- | --- | --- |
| **1JJI-*A. fulgidus*** |  | 25.92 | 27.91 | 38.80 | 51.75 | 26.72 | 28.17 | 34.87 | 31.67 | 15.29 |
| **1JKM-*B. subtilis*** | 25.92 |  | 24.78 | 24.35 | 28 | 46.66 | 23.36 | 24.21 | 23.64 | 14.20 |
| **1LZL-*Rhodococcus* sp.** | 27.91 | 24.78 |  | 31.57 | 28.70 | 25.64 | 28.22 | 25.68 | 24.84 | 15.69 |
| **1QZ3-*A. acidocaldarius*** | 38.80 | 24.35 | 31.57 |  | 42.22 | 24.63 | 33.85 | 31.46 | 30.43 | 15.88 |
| **3ZWQ-*P. calidifontis*** | 51.75 | 28.00 | 28.70 | 42.22 |  | 27.53 | 33.5 | 34.05 | 36.13 | 17.10 |
| **4J7A-Metagenomic** | 26.72 | 46.66 | 25.64 | 24.63 | 27.53 |  | 21.55 | 22.12 | 23.34 | 11.87 |
| **4OU4-*P. putida*** | 28.17 | 23.36 | 28.22 | 33.85 | 33.50 | 21.55 |  | 33.22 | 39.06 | 15.38 |
| **4WY5-*R. meihei*** | 34.87 | 24.21 | 25.68 | 31.46 | 34.05 | 22.12 | 33.22 |  | 40.18 | 15.63 |
| **4WY8-*R. meihei*** | 31.60 | 23.64 | 24.84 | 30.43 | 36.13 | 23.34 | 39.06 | 40.18 |  | 16.91 |
| **BaEstB** | 15.29 | 14.20 | 15.69 | 15.88 | 17.10 | 11.87 | 15.38 | 15.63 | 16.91 |  |
